# Efficient Enrichment of Docosahexaenoic Acid (DHA) in Mother’s Milk and in the Brain and Retina of the Offspring by Lysophosphatidylcholine (LPC)-DHA in the Maternal Diet

**DOI:** 10.3390/nu17111864

**Published:** 2025-05-29

**Authors:** Poorna C. R. Yalagala, Dhavamani Sugasini, Sutape Chantapim, Karyna Caal, Haijing Sun, Sofia Nicastro, Robert M. Sargis, Brigid Gregg, Papasani V. Subbaiah

**Affiliations:** 1 Division of Endocrinology, Diabetes and Metabolism, Department of Medicine, University of Illinois Chicago, Chicago, IL 60612, USA; yalagala@uic.edu (P.C.R.Y.); sugasini@uic.edu (D.S.); rsargis@uic.edu (R.M.S.); 2 Department of Pediatrics, University of Michigan, Ann Arbor, MI 48109, USA; kcaal@umich.edu (K.C.); haijings@med.umich.edu (H.S.); greggb@med.umich.edu (B.G.); 3 Jesse Brown VA Medical Center, Chicago, IL 60612, USA; 4 Department and Nutritional Sciences, School of Public Health, University of Michigan, Ann Arbor, MI 48109, USA

**Keywords:** dietary omega 3 fatty acid, docosahexaenoic acid, lysophosphatidylcholine, triacylglycerol, milk, brain, retina, liver, adipose tissue

## Abstract

**Background:** Docosahexaenoic acid (DHA) is the most important fatty acid (FA) for the development and function of brain and retina. Mother’s milk is the predominant source of DHA for the baby’s postnatal life, and the omega 3 FA content of a mother’s diet is highly correlated with the cognitive and visual functions of the infant. However, clinical trials aimed at increasing the DHA content of mother’s milk and thereby improving infant cognitive function have been inconclusive. **Methods**: In this study, we tested the hypothesis that the molecular form of dietary DHA is important in enriching DHA in mother’s milk as well as in pup tissues. Lactating dams were fed defined diets containing DHA either in the form of triacylglycerol (TAG) or lysophosphatidylcholine (LPC), and the FA composition of mother’s milk and pup tissues was determined on postnatal day 16. **Results:** The results showed that LPC-DHA was 5-fold more efficient than TAG-DHA in enriching milk DHA. Moreover, DHA content was increased by 31% in the brain, 56% in the retina, and 14% in the liver of the pups by LPC-DHA in the maternal diet, whereas no increases were observed with TAG-DHA. The DHA content of the pup adipose tissue, however, was increased equally by the DHA supplements. **Conclusions:** These results show that dietary LPC-DHA is a promising new strategy to increase milk DHA content and to potentially improve brain and retinal health in infants. This strategy may be more important in the care of premature infants who miss the critical prenatal period of DHA accretion in the last trimester of pregnancy.

## 1. Introduction

DHA (docosahexaenoic acid) is the most abundant polyunsaturated fatty acid (PUFA) in the brain and retina of mammals and is critical for the development of these organs in early life. It plays a vital role in synaptogenesis, membrane function, and myelination, as well as in visual function. The intense brain growth spurt, which occurs in humans in the last trimester of pregnancy and continues until 2 years of age, corresponds to the enhanced accretion of DHA by the infant brain [1]. Mother’s milk is the predominant source of DHA in early postnatal life, and therefore the omega 3 fatty acid (FA) content of mother’s diet greatly influences the brain development of the infant. Epidemiologic studies show a positive correlation of omega 3 FA consumption by the mother with the cognitive function of the infant, as well as with lower levels of asthma, allergy, and eczema [2]. Experimental deficiency of omega 3 FA during pregnancy and lactation in mice results in functional deficits in vision, learning, and memory and motor development in the offspring [3]. Furthermore, supplementation of mother’s diet with DHA during gestation increases the brain DHA content of the offspring and improves neuroplasticity and brain function in mice [4,5]. The maternal milk DHA content was shown to predict cognitive test scores of 15 year-old olds more consistently than other factors such as per capita gross domestic product or educational expenses per pupil [6]. On the other hand, elevated content of omega 6 FA including linoleic acid (LA) and arachidonic acid (ARA) in the milk was negatively associated with developmental health outcomes and adiposity indices [2], although ARA is also required for optimal brain development [7]. Interestingly, the ARA content of mother’s milk is less variable than that of DHA, which varies more widely depending upon the dietary habits, culture, and lifestyle [8,9]. Therefore, dietary supplementation of DHA to the mother is an important strategy to optimize the development of the infant. DHA supplementation is even more important for pre-term infants since they do not acquire DHA during the critical period of the last trimester of pregnancy. Despite the epidemiologic evidence, however, randomized controlled trials of supplementation of PUFA to breast feeding mothers aimed at improving infant growth, brain development, visual acuity, or allergies have yielded inconclusive results [10,11]. One possible reason for this failure is the inadequate enrichment of mother’s milk DHA by the type of supplements used. In these studies, DHA supplements were almost invariably in the form of triacylglycerol (TAG) (ex: fish oil, algal oil). In contrast, the epidemiologic studies reflect the maternal consumption of sea food where part of the DHA is present in the form of phospholipids, which has been suggested to be more bioavailable than the DHA from TAG [12]. We recently demonstrated that DHA in the form of lysophoshatidylcholine (LPC) is vastly superior to TAG-DHA or free DHA in enriching brain and retinal DHA and in improving memory and visual functions in adult mice and rats [13,14,15]. This is because of the presence of a transporter specific for LPC-DHA at the blood–brain barrier and blood retina barrier [16]. In this study, we tested the hypothesis that supplementing the maternal diet of lactating mice with LPC-omega 3 FA would increase the milk DHA content and consequently enrich the brain and retinal DHA of the pups.

## 2. Methods

### 2.1. Diets, Animals, and Procedures

All studies in animals described here were approved by the Animal Care committee at the University of Michigan (Protocol # PRO 00012189), on 8 October 2024, and expires on 8 October 2027. DHA-enriched diets were prepared by Dyets, Inc. (Bethlehem, PA, USA) by incorporating either TAG-DHA from algal oil (DSM Nutritional Products, Columbia, MD, USA) or synthetic LPC-DHA (sn-1-DHA LPC, Avanti Polar Lipids, Birmingham, AL, USA) into AIN93G diet formulation. The control diet contained 7% corn oil as the fat source. Experimental diets contained either TAG-DHA or LPC-DHA at a concentration of 1 µmole of DHA/g diet. This dose was selected based on our previous studies in mice [14,17,18]. The total fat content was kept at 7% in all diets, and the animals had full access to water and food throughout.

Male and female C57BL/6J mice were purchased from Jackson Laboratories (RRID: IMSR_JAX:000664 https://scicrunch.org/resolver/IMSR_JAX:000664; accessed on 1 March 2024). Mice were housed under standard conditions with 12 h light/dark cycles and were given ad libitum access to food. All animal procedures in this study were approved by the University of Michigan Institutional Animal Care and Use Committee.

All dams were maintained on 5L0D standard mouse diet (LabDiet, St. Louis, MO, USA) throughout mating and gestation. Mice were bred under standard conditions, using a breeding trio of 2 females and 1 male per cage. A total of 30 females went through 2 rounds of breeding. This resulted in 44 litters of pups with 14 control litters and 15 each of LPC-DHA and TAG-DHA. Average litter sizes ranged from 6 to 7.1 between the groups. On the day of birth, referred to in this study as postnatal day 1 (PND1), dams were switched to the DHA diets based on AIN93G formulation. They were randomly placed into three groups (*n* = 6 in each group): one receiving control diet (no DHA), a second receiving LPC-DHA enriched chow, and a third receiving TAG-DHA enriched chow during the postnatal lactation window. The sample size was determined from our previous studies on the absorption and tissue distribution of DHA compounds [14,17,18].

#### 2.1.1. Milk Collection

Milk was collected from the dams on PND 16. Only second time mothers were used for milk collection as full maturation of the mouse mammary gland may not occur until the second litter. Milk collected from six dams was used for each treatment. The dam was separated from its pups for two hours, during which the pups were euthanized for tissue collection, and the dam had full access to food and water. After a two-hour separation, the dam was placed under general anesthesia by injecting ketamine and xylazine (5 µL/g) into the hind leg muscle. Once the dam stopped moving, 0.5 U/µL (2 units total) of oxytocin was injected intramuscularly into the fore leg. An empty 1.5 mL Eppendorf tube was weighed and used to collect milk using a 10 µL pipette. Each nipple was milked until the milk had a watery consistency. The tube was then weighed again; 20 µL milk was aliquoted for downstream analysis and frozen at −80 °C.

#### 2.1.2. Tissue Collection

Dams and pups were euthanized at PND 16 via isoflurane inhalation. Pup brain, retina, liver, and inguinal white adipose tissue (IWAT) were collected. The tissues were quick frozen in liquid N_2_ and stored at −80 °C until the lipid analysis.

### 2.2. Analysis of Fatty Acids

Total lipids were extracted from all tissues by the Bligh and Dyer method [19]. The preparation of methyl esters of fatty acids and GC/MS analysis were performed as described previously [15] after including 17:0 PC and 15:0 TAG as internal standards. The individual fatty acids values are expressed as % of total fatty acids. Statistical significance among groups was determined by one-way ANOVA, correcting for multiple comparisons, using Tukey test (Graphpad Prism 10 software). Except where indicated, all analyses were performed on *n* = 6 per group.

## 3. Results

### 3.1. Effect of Dietary TAG-DHA and LPC-DHA on Fatty Acid Composition of Milk

**Figure 1:**
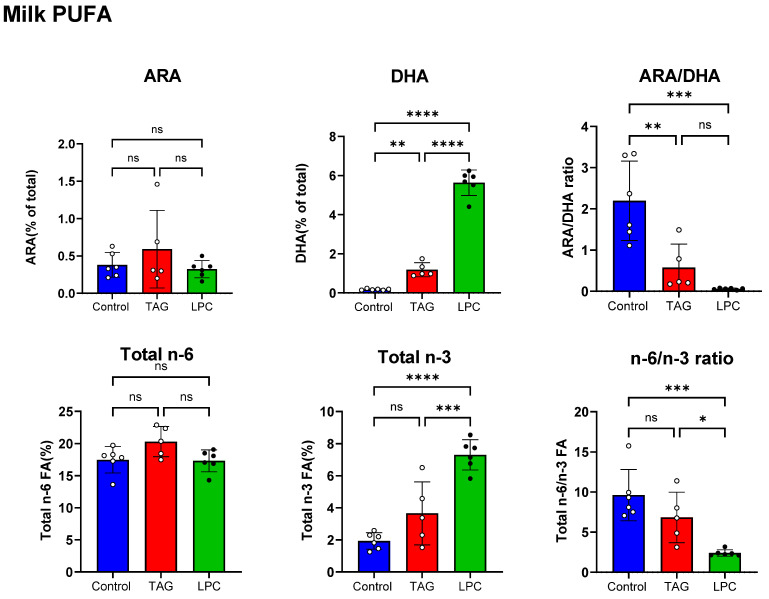
Effect of molecular form of dietary DHA on milk PUFA composition. Nursing dams were fed ad libitum from PND1 to PND16 either control diet (AIN93G diet with no DHA) or AIN93G diets supplemented with I µmole of DHA/g in form of TAG or LPC. Milk was collected on PND 16 and was analyzed for fatty acid composition by GC/MS. Total FA composition is shown in Table S1, Supplementary Materials. Data shown are mean ± SD of *n* = 6 samples per group. Statistical significance was determined by one-way ANOVA using Graphpad Prism 10 software. * *p* < 0.05; ** *p* < 0.01; *** *p* < 0.001; **** *p* < 0.0001; ns: not significant.

Lactating dams were fed the control diet (no DHA) or a diet enriched in either TAG-DHA or LPC-DHA from PND1 to PND16, and the milk was collected on PND16 as described above. The body weights and body fat composition of the dams were not affected by either DHA diet. Figure 1 (top panel) shows the DHA and ARA contents as well as the ARA/DHA ratios in the milk. The total fatty acid composition is shown in Supplementary Data, Table S1. Both DHA diets increased the DHA content of the milk significantly compared to the control. The TAG diet increased the DHA by 6.6-fold (to 1.19% of total FA) compared to the control diet (0.18% of total FA), comparable to the results reported by Oosting et al. [20], whereas the LPC-DHA diet increased it by 31-fold (to 5.63% of total FA). The ARA content of the milk, however, was not significantly affected by either TAG-DHA or LPC-DHA diets. The ARA/DHA ratio was significantly reduced by both the DHA diets, but the decrease by LPC-DHA (−97%) was greater than that by TAG-DHA (−74%). The increase in DHA was apparently at the expense of the saturated fatty acids in the milk (Table S1, Supplementary Materials), although the decrease in these fatty acids from the control did not reach statistical significance (ANOVA).

We then analyzed the total *n*-6 and total *n*-3 FA, as well as their ratios in milk (Figure 1, bottom panel). The total *n*-6 FA (18:2, 18:3, 20:2, 20:3, 20:4, 22:4, and 22:5) was not altered by either DHA supplement. The total *n*-3 FA (18:3, 20:5, 22:3, 22:5, and 22:6) were increased by both the DHA diets compared to the control diet, but only the increase by the LPC-DHA diet was statistically significant. The *n*-6 FA/*n*-3 FA ratio was reduced by both supplements (29% by TAG-DHA, and 72% by LPC-DHA), but only the decrease by LPC-DHA was statistically significant.

### 3.2. Effect of TAG-DHA and LPC-DHA in Maternal Diet on Pup Brain Fatty Acid Composition

**Figure 2:**
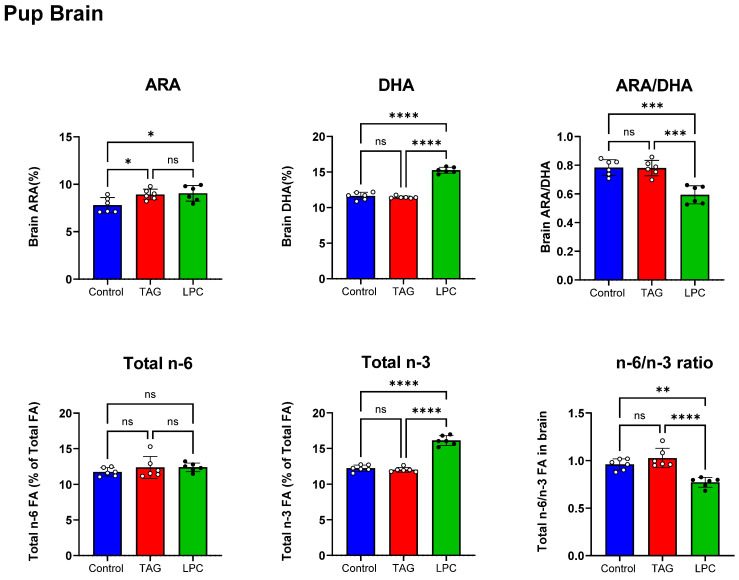
Effect of molecular form of DHA in maternal diet on PUFA composition of brain lipids in offspring. Brains from PND16 male pups of dams fed control, TAG-DHA, or LPC-DHA diets were analyzed for fatty acid composition by GC/MS. Values shown are mean ± SD of *n* = 6 samples per group. Total fatty acid composition of brain is shown in Supplementary Materials, Table S2. Statistical analysis and *p* values are as described in Figure 1; ns: not significant.

We next determined the effect of maternal DHA supplements on the fatty acid composition of the pup tissues. The body weights of the pups (PND 16) were not affected by the DHA supplements in the maternal diet. As shown in Figure 2 (top panel), the brain DHA content of the pups was significantly increased in the LPC-DHA group (+31%) compared to the control, but there was no increase in the TAG-DHA group. This shows that the modest increase in milk DHA by the maternal TAG DHA diet was not sufficient to increase the brain DHA content in the pups. Interestingly, the percentage of ARA in the brain lipids was increased in both groups, although the levels of ARA and other *n*-6 FA were not changed significantly in the milk by the supplements. The ARA/DHA ratio, however, was significantly decreased only in the LPC-DHA group (−24% from control). There were significant decreases in 18:1 (*n*-9), 18:1 (*n*-7), 20:0, 20:1 (*n*-9), and 22:0 fatty acids in the LPC-DHA group to compensate for the increase in DHA (Table S2, Supplementary Materials).

The total *n*-6 and *n*-3 FA levels were then determined in the pup brains (Figure 2, bottom panel). Although the brain ARA concentration was increased by both the experimental diets, there was no significant effect on total *n*-6 FA. The total *n*-3 FA was not affected by the TAG-DHA diet but was significantly increased by the LPC-DHA diet. Consequently, the *n*-6/*n*-3 ratio of pup brains was significantly decreased by LPC-DHA (−20% from control), but not by TAG-DHA in the maternal diet.

**Figure 3:**
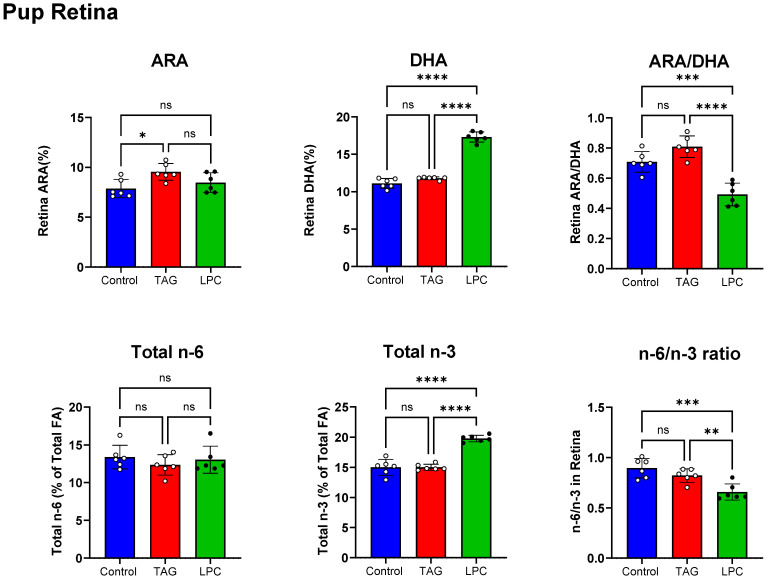
Effect of molecular form of maternal dietary DHA on retinal PUFA composition in offspring. Retina samples from PND16 male pups from dams fed control, TAG-DHA, and LPC-DHA diets were analyzed for fatty acid composition by GC/MS. Retinas were collected by enucleating eye, making a cut at cornea–sclera junction, and then removing lens and vitreous body under dissection scope. Two retinas from each pup were pooled and analyzed as a single sample. Values shown are mean ± SD of *n* = 6 samples per group. Total FA composition of retina is shown in Supplementary Materials, Table S3. Statistical analysis and *p* values are as described in Figure 1; ns: not significant.

Figure 3 (top panel) shows the effect of maternal dietary DHA on retinal DHA and ARA in PND 16 pups. Like in the brain, the DHA content of pup retina increased significantly (+56% compared to the control) in the LPC-DHA group, but there was no effect in the TAG-DHA group. The percentage of ARA increased in the TAG-DHA group (+21%, compared to control group), but not in the LPC-DHA group. The ARA/DHA ratio significantly decreased in the LPC-DHA group (−30%, compared to the control), but not in the TAG-DHA group. The ARA/DHA ratio was 40% lower in the retina of the LPC-DHA pups compared to the TAG-DHA pups. The total *n*-6 and total *n*-3 FA levels in pup retinas from the three groups are shown in the bottom panel of Figure 3. Like the brain, the retinal *n*-6 FA levels were unaffected by either LPC-DHA or TAG-DHA in the maternal diet. The *n*-3 FA levels of pup retina were significantly increased by LPC-DHA but not by TAG-DHA in the maternal diet. The ratio of *n*-6/*n*-3 was decreased by the LPC diet (−26% compared to control), but not by the TAG diet.

**Figure 4:**
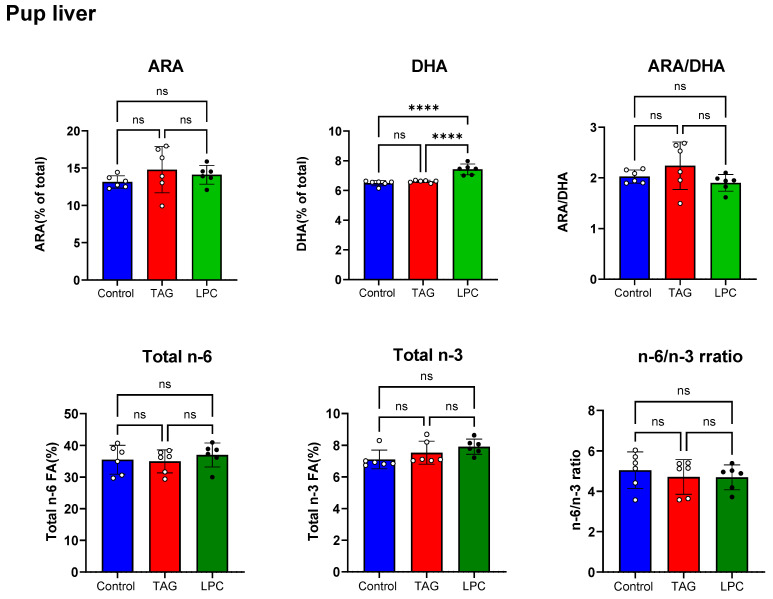
Effect of TAG-DHA and LPC-DHA in maternal diet on liver PUFA composition in offspring. Livers from PND 16 male pups of dams fed control, TAG-DHA, or LPC-DHA were analyzed for fatty acid composition by GC/MS. Values shown are mean ± SD of *n* = 6 samples per group. Total FA composition of pup livers is shown in Supplementary Materials, Table S4. Statistical analysis and *p* values are as described in Figure 1; ns: not significant.

We also measured the effect of maternal dietary DHA on the levels of DHA and ARA in the livers of pups on PND 16 (Figure 4, top panel). Only the LPC-DHA group showed a significant increase in liver DHA (+14%), compared to the control group. The ARA levels as well as the ARA/DHA ratios of pup livers were unaffected by either DHA supplement in the maternal diet. Our previous studies in adult mice and rats also showed that LPC-DHA in diet enriched the liver DHA more efficiently than either TAG DGA free DHA [13,14,15]. Neither TAG-DHA nor LPC-DHA in the maternal diets increased the total *n*-6 or total *n*-3 levels (Figure 4, bottom panel). The *n*-6/*n*-3 ratios were not affected by either diet.

**Figure 5:**
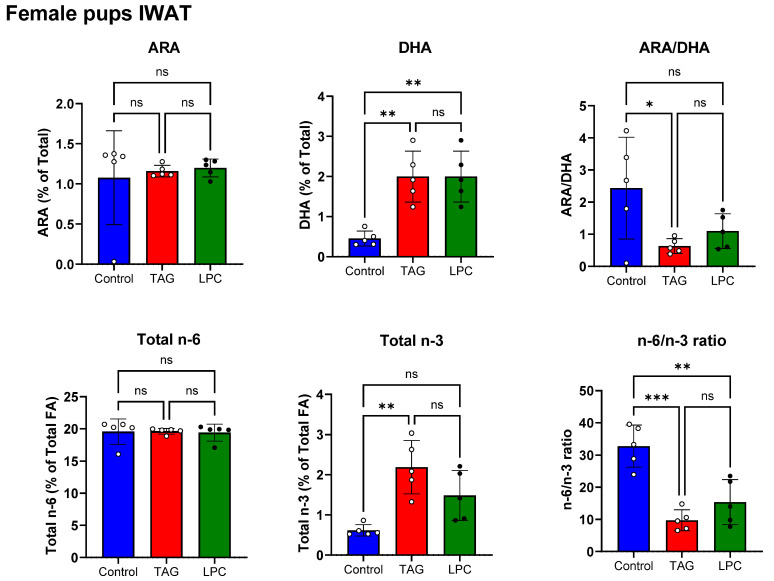
Effect of molecular form dietary DHA in maternal diet on IWAT PUFA composition in female offspring. IWAT samples from female pups (PND16) of dams fed control, TAG-DHA, or LPC-DHA diets were analyzed for fatty acid composition by GC/MS. Values shown are mean ± SD of *n* = 5 samples per group. Total FA composition is shown in Supplementary Materials, Table S5. Statistical analysis and *p* values are as described in Figure 1; ns: not significant.

Figure 5 shows the percentages of DHA and ARA in IWAT of the female pups on PND 16 (top panel). In contrast to other tissues, the adipose tissue of the pups acquired similar amounts of DHA in the TAG-DHA group and the LPC-DHA group. It should be pointed out that the DHA content of the milk in the TAG group is only about 20% of the level found in the LPC-DHA group, and therefore TAG DHA in the maternal diet increases the DHA in adipose tissue of the pups more efficiently, compared to the DHA from dietary LPC. In contrast, DHA in maternal dietary LPC-DHA is directed more towards brain, retina, and liver. This is similar to the results we found in adult mice and rats after feeding diets enriched with LPC-DHA and TAG-DHA [13,14]. The ARA levels in IWAT were not significantly affected by either form of DHA in the maternal diet. The decrease in ARA/DHA ratio was statistically significant in the TAG-DHA group, but not in the LPC-DHA group.

The bottom panel of Figure 5 shows the total *n*-6 and total *n*-3 FA levels in IWAT of female pups. The total *n*-6 FA was not affected by either form of DHA in the maternal diet. The total *n*-3 FA increased significantly by the TAG-DHA diet but not by the LPC-DHA diet. The *n*-6/*n*-3 ratio of IWAT was decreased by both diets but only the effect of TAG-DHA was significant.

**Figure 6:**
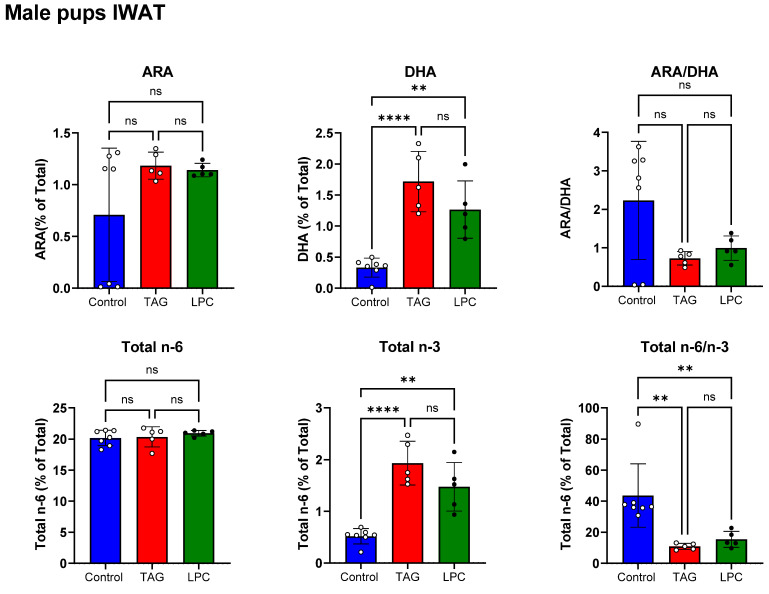
Effect of molecular form dietary DHA in maternal diet on IWAT PUFA composition in male offspring. IWAT samples from male pups (PND16) of dams fed control, TAG-DHA, or LPC-DHA diets were analyzed for fatty acid composition by GC/MS. Values shown are mean ± SD of *n* = 7 samples in control, and *n* = 5 samples in TAG-DHA and LPC-DHA groups. Total FA composition is shown in Supplementary Materials, Table S6. Statistical analysis and *p* values are as described in Figure 1; ns: not significant.

Figure 6 (top panel) shows the ARA and DHA percentages in IWAT of male pups on PND 16. Like the female pups, the ARA content was not affected by either maternal DHA diet. The adipose DHA was increased by both diets, although the TAG group showed a greater increase. Unlike the female pups, however, the decrease in ARA/DHA ratio in male pups was not statistically significant in either diet group.

The bottom panel of Figure 6 shows the total *n*-6 and total *n*-3 FA in IWAT of male pups. The *n*-6 FA content was not affected by either DHA diet, but the total *n*-3 FA levels were significantly increased by both diets. The ratio of *n*-6/*n*-3 FA was significantly decreased by both diets compared to the control.

## 4. Discussion

The major objective of this study was to investigate whether the DHA content of milk can be increased by altering the molecular carrier of DHA in the maternal diet. Our results show that dietary DHA in the form of LPC was 5-fold more efficient than the currently used TAG-DHA (fish oil, algal oil) in enriching the milk DHA. To our knowledge, this level of DHA enrichment in milk (5.56% of total FA) by diet has not been reported previously in experimental animals or humans. More importantly, this enrichment of milk DHA by dietary LPC-DHA resulted in a significant increase in the DHA content of the brain and retina in the pups, whereas TAG-DHA was ineffective at the same dose. The DHA concentration found in the pup brain of the LPC-DHA group is comparable to that achieved by feeding milk from transgenic dams, who secrete phospholipid DHA-enriched milk [21]. There are several possible reasons for the more efficient DHA enrichment of milk as well as the offspring brain and retina by maternal dietary LPC-DHA compared to TAG-DHA. First, LPC-DHA, which does not need enzymatic hydrolysis, is more easily absorbed compared to TAG-DHA, hence the incorporation into the maternal tissues is more efficient. Secondly, dietary LPC-DHA may increase the amount of phospholipid DHA in milk. It has been reported that, although the phospholipid content of the milk is around 2% of the total fat, 11% of DHA in the milk is present in the phospholipids. Therefore, increasing the phospholipid percentage even modestly can increase the milk DHA content significantly. Unfortunately, we have not been able to determine the relative distribution of DHA between the phospholipids and TAG of milk to test this possibility because of the limitation of the sample volume. Thirdly, it is possible that the DHA from dietary TAG is first incorporated into other tissues such as peripheral adipose tissues and heart before appearing in the milk, whereas the phospholipid DHA is more directly incorporated into the milk lipids. Valenzuela et al. [22] reported that dietary DHA in the form of phospholipid and monoacylglycerol increased the milk DHA content more than either the TAG or ethyl ester forms of DHA in rats. Similarly in transgenic mice that convert endogenous *n*-6 FA to *n*-3 FA, the bulk of DHA and EPA appear in the phospholipids of the milk and consequently enrich brain DHA in the pups [21]. It has been shown in humans that about 20% of DHA from dietary TAG is incorporated into the milk within 48 h [23], but the majority of milk DHA comes from tissue reserves, mostly the adipose tissue. It is possible that DHA from dietary LPC is incorporated at a higher rate into milk, since unlike dietary TAG, it is not directed first to the adipose tissue [13]. Time course studies using labeled dietary TAG and LPC need to be performed to investigate this possibility.

Our study also shows that increasing the milk DHA through dietary LPC-DHA has significant effects on lipid profiles of the pup tissues. Whereas the brain and retinal DHA contents in the pups were increased markedly by LPC-DHA in the maternal diet, TAG-DHA of the maternal diet leads to preferential enrichment of adipose tissue, but no enrichment of brain or retina. As is consistent with this observation, a recent study in mice [24] reported that even a high dose of fish oil (1.36 g/kg of EPA + DHA) during pregnancy and lactation did not increase the DHA content of retina in the offspring. Although the dietary DHA is in the form of sn-1 DHA LPC, it is likely that it is released as free DHA in the tissues and incorporated into phospholipids in its more natural sn-2 position by the normal metabolic pathways. This is supported by our observation that the major increase in brain DHA occurs in PE, rather than PC, after feeding sn-1 DHA LPC [15].

Our previous studies in adult mice and rats showed that although the total absorption of free DHA and LPC-DHA is similar, the tissue distribution of the absorbed DHA is markedly different. We showed that DHA from dietary TAG and free DHA is absorbed as TAG in chylomicrons and is incorporated predominantly into adipose tissue and heart during the passage of chylomicrons through these tissues by the actions of lipoprotein lipase. In contrast, the DHA from dietary LPC is absorbed as phospholipid, and is ultimately more available for uptake by brain and retina in the form of LPC-DHA [13,14,15,18]. In the current study, we find that whereas the DHA content of milk in the TAG-DHA-fed dams is only 20% of that in the LPC-DHA-fed dams, the adipose tissue enrichment is comparable in both groups or even higher in the pups from the TAG-DHA group. This also suggests the possible enrichment of milk phospholipids in the LPC-DHA group, since the dietary phospholipid DHA is less likely to enrich adipose tissue DHA of the mother, based on our previous studies in adult mice and rats [13,15].

Despite the extensive support from the observational, epidemiologic, and experimental data for the benefits of increasing milk DHA, randomized controlled trials using dietary fish oil to increase milk DHA content and thereby improve the developmental outcomes in infants have produced inconclusive results [10,11]. Several studies showed that human milk DHA can be increased modestly by dietary TAG-DHA [23,25,26,27], but this does not result in a meaningful improvement in the visual or cognitive functions of the infant. This may be because fish oil, at tolerable doses in the maternal diet, does not sufficiently enrich milk DHA to improve the brain and retinal function in the infants. Although the world-wide average of human milk values for DHA is 0.32 ± 0.22% [8], the optimal DHA content for infant development was proposed to be 1% of total FA [28] corresponding to a maternal erythrocyte DHA content of 8%. It is also important to replenish the maternal DHA reserves which are depleted during the gestation and lactation periods [28]. Furthermore, the DHA content of the milk decreases significantly within 30 days of birth, possibly as a consequence of depletion of maternal reserves. For these reasons, the dietary DHA level needs to be increased more than the currently recommended levels of 200 mg/day for the lactating women. However, it takes large amounts of fish oil to achieve these objectives. For example, it has been calculated that 1 g of DHA (equal to 3–4 g of fish oil) per day is required to increase the milk DHA content by 0.38% [29]. Another study reported that feeding 10 g of fish oil per day increased the milk DHA from 0.1% to 0.8% of total fatty acids [30]. Since consuming such large amounts of fish oil for long periods of time is not feasible, LPC-DHA, which is 5-fold more efficient than TAG-DHA in enriching milk DHA, would be preferable. Moreover, the added benefits to the mother in replenishing the DHA depleted during gestation and lactation, and potentially in prevention of postpartum depression [31], warrant further investigation of the potential benefit of dietary LPC-DHA for lactating mothers.

It has been shown that in addition to DHA, the ARA content of breast milk is important for the optimal development of the infant [7]. Since the dietary DHA, in general, replaces ARA in the tissues [13,15,32], it is important to determine the effect of DHA enrichment on the ARA content of the milk. We found that despite a 30-fold increase in milk DHA content by maternal LPC-DHA diet, the concentration of milk ARA was not affected. This is consistent with human studies which showed that maternal DHA content does not affect the milk ARA content [2,8,25]. It is also noteworthy that although there was a significant decrease in the ARA/DHA ratio and the *n*-6/*n*-3 FA ratio in most pup tissues, this decrease was solely due to an increase in DHA and not due to a decrease in ARA.

There are a few limitations of our study: (1) We have not measured the dose-dependent effects of dietary LPC-DHA on the enrichment of pup tissues. (2) We did not determine the functional effects of DHA enrichment in the pup brain or retina. Since excess DHA in these tissues may not be desirable, a correlation of the dose of LPC-DHA in a mother’s diet with the functional effects on pup brain and retina is needed. (3) We have not measured the incorporation of dietary DHA in the tissues of the mother. (4) We have analyzed the milk samples only at one time point (PND 16). (5) The distribution of DHA between the TAG and phospholipids of milk could not be determined because of the limitation of milk volume. Nevertheless, these results may have practical clinical applications since the tissue distribution and metabolism of DHA are similar in humans and rodents [15,24,33,34]. The contribution of milk DHA to the tissues of the baby is especially important for pre-term infants who miss the intrauterine accretion of DHA, which occurs mostly in the last trimester of pregnancy [1]. Therefore, it would be beneficial for the DHA content of mother’s milk to be much higher for these infants because development of their brain and retina is dependent on the supply of DHA from the milk. In addition, these infants are more susceptible to retinopathy of prematurity (ROP) and neurodevelopmental diseases [35] because of the depletion of DHA due to increased oxidative damage in the incubator/isolette in the neonatal intensive care setting. Studies in mice have demonstrated that feeding very high amounts of TAG-DHA (2% DHA w/w in the diet) to nursing dams increased the retinal DHA to about 18% of total FA (levels similar to those achieved in the current study with a 60-fold lower dose of LPC-DHA), and reduced the pathological angiogenesis in the retina of the pups subjected to oxygen-induced retinopathy [36]. However, it is unrealistic to feed humans large amounts of TAG-DHA equivalent to those fed to mice for the prevention of ROP (about 40 mL fish oil or 25 mL algal oil per day for a 60 kg woman). Therefore, dietary LPC-DHA, which is required in a much smaller dose (about 200 mg DHA per day), is a promising alternative. It is also possible that including LPC-DHA in the infant formula would be more effective since DHA from the phospholipids has been shown to be absorbed more efficiently in pre-term infants, compared to TAG-DHA [37], and LPC-DHA would not require enzymatic digestion before absorption.

## 5. Conclusions

In this study, we have provided proof of concept that a dietary LPC-DHA supplementation strategy in lactating mice can efficiently enhance milk DHA concentration and consequently increase brain and retinal DHA levels in their offspring. This could pave the way for future translational studies to investigate the implications for lactating women. In addition, life-course studies on the effects of this supplementation on offspring outcomes will also be informative.

## Data Availability

The original contributions presented in this study are included in the article/Supplementary Material. Further inquiries can be directed to the corresponding author.

## Supplementary Materials

The following supporting information can be downloaded at: https://www.mdpi.com/article/10.3390/nu17111864/s1, Table S1: Milk fatty acid composition; Table S2: Pup brain fatty acid composition; Table S3: Pup retina fatty acid composition; Table S4: Pup liver fatty acid composition; Table S5: FA composition female pups IWAT; Table S6: FA composition of male pups IWAT.
