# Supplementary material for: Efficient Enrichment of Docosahexaenoic Acid (DHA) in Mother’s Milk and in the Brain and Retina of the Offspring by Lysophosphatidylcholine (LPC)-DHA in the Maternal Diet
Source: Nutrients. 2025 May 29;17(11):1864. doi: 10.3390/nu17111864 (PMC12157575; doi:10.3390/nu17111864)
Supplement: Supplementary file 1 [file nutrients-17-01864-s001.zip › nutrients-3626097-supplementary.pdf]

Supplementary Materials, Table -S1

Milk Fatty Acid Composition (% of Total FA) (Mean ± SD)

FA	Control diet (n=4)			TAG-DHA diet (n=5)			LPC-DHA diet (n=6)		
12:0	5.37	±	0.97	5.47	±	0.59	4.64	±	1.06
14:0	9.83	±	0.22	9.31	±	1.80	8.27	±	1.86
16:0	29.94	±	3.82	24.84	±	3.17	24.74	±	2.91
16:1(n-7)	2.15	±	1.04	2.00	±	0.88	2.43	±	0.47
17:1(n-9?)	3.10	±	3.47	2.13	±	2.81	4.69	±	0.92
18:0	5.06	±	1.58	3.02	±	2.47	4.38	±	2.34
18:1(n-9)	19.84	±	1.56	23.11	±	1.35	21.21	±	3.68
18:1(n-7)	0.72	±	1.04	1.36	±	1.14	2.03	±	0.91
18:2 (n-6)	14.33	±	1.84	14.95	±	2.74	13.88	±	1.48
18:3 (n-6)	0.36	±	0.18	0.58	±	0.37	0.38	±	0.15
18:3 (n-3)	0.29	±	0.29	0.27	±	0.19	0.22	±	0.14
20:0	1.98	±	0.52	2.56	±	0.73	1.84	±	0.68
20:1 (n-9)	0.46	±	0.21	0.66	±	0.62	0.34	±	0.36
20:2 (n-6)	1.12	±	0.27	1.16	±	0.50	0.63	±	0.32
20:3 (n-6)	0.49	±	0.34	0.88	±	0.71	0.55	±	0.27
20:4 (n-6)	0.40	±	0.21	0.59	±	0.52	0.32	±	0.11
22:0	0.48	±	0.13	0.60	±	0.17	0.39	±	0.17
20:5 (n-3)	0.47	±	0.03	0.86	±	0.80	0.62	±	0.53
22:2 (n-6)	0.61	±	0.27	0.74	±	0.67	0.67	±	0.36
22:3 (n-3)	0.53	±	0.30	0.56	±	0.58	0.39	±	0.10
22:4 (n-6)	0.71	±	0.57	0.83	±	0.54	0.46	±	0.29
22:5 (n-6)	0.20	±	0.10	0.56	±	0.44	0.44	±	0.21
22:5 (n-3)	0.43	±	0.20	0.79	±	0.72	0.45	±	0.24
22:6 (n-3)	0.17	±	0.02 [a]	1.19	±	0.36 [b]	5.63	±	0.65 [c]
24:1(n-9)	0.56	±	0.14	0.70	±	0.59	0.22	±	0.14
16:0 DMA	0.16	±	0.06	0.19	±	0.14	0.12	±	0.05
18:0 DMA	0.55	±	0.21	0.39	±	0.42	0.31	±	0.19
18:1 DMA	0.20	±	0.03	0.26	±	0.34	0.15	±	0.08

Values without a common superscript are significantly different from each other (ANOVA)

DMA: Dimethylacetal

Supplementary Materials, Table-S2

Pup Brain Fatty Acid Composition (% of Total FA) (Mean ± SD)

FA	Control diet (n=6)	TAG-DHA diet (n=6)	LPC-DHA diet (n=6)
12:0	0.03 ± 0.02	0.04 ± 0.04	0.10 ± 0.07
14:0	0.03 ± 0.02	0.07 ± 0.09	0.06 ± 0.07
16:0	23.01 ± 1.36	22.21 ± 1.47	23.68 ± 2.32
16:1(n-7)	0.46 ± 0.31 [a]	0.70 ± 0.10 [ab]	0.88 ± 0.32 [bc]
17:1(n-9?)	0.06 ± 0.06 [a]	0.03 ± 0.02 [a]	0.71 ± 0.44[b]
18:0	20.90 ± 0.66	20.59 ± 1.06	20.69 ± 1.05
18:1(n-9)	21.83 ± 1.45 [a]	22.19 ± 0.56 [a]	17.12 ± 3.68 [b]
18:1(n-7)	4.99 ± 0.35 [a]	5.03 ± 0.21 [a]	4.20 ± 0.22 [b]
18:2 (n-6)	1.09 ± 0.11	1.18 ± 0.38	1.44 ± 0.69
18:3 (n-6)	0.02 ± 0.02	0.04 ± 0.04	0.09 ± 0.10
18:3 (n-3)	0.03 ± 0.02	0.07 ± 0.08	0.23 ± 0.43
20:0	0.60 ± 0.11 [a]	0.63 ± 0.11[ab]	0.44 ± 0.12 [ac]
20:1 (n-9)	3.24 ± 0.79 [a]	3.52 ± 0.36 [a]	1.90 ± 1.08 [b]
20:2 (n-6)	0.95 ± 0.15	1.48 ± 0.80	0.89 ± 0.29
20:3 (n-6)	0.29 ± 0.18	0.33 ± 0.43	0.42 ± 0.30
20:4 (n-6)	9.12 ± 0.69	8.93 ± 0.55	9.05 ± 0.83
22:0	0.05 ± 0.05	0.05 ± 0.03	0.21 ± 0.17
20:5 (n-3)	0.41 ± 0.04	0.31 ± 0.20	0.34 ± 0.18
22:2 (n-6)	0.07 ± 0.03	0.12 ± 0.12	0.44 ± 0.48
22:4 (n-6)	0.11 ± 0.05	0.10 ± 0.07	0.22 ± 0.26
22:5 (n-6)	0.18 ± 0.15	0.32 ± 0.15	0.31 ± 0.16
22:5 (n-3)	0.18 ± 0.15	0.20 ± 0.12	0.29 ± 0.18
22:6 (n-3)	11.64 ± 0.52 [a]	11.45 ± 0.17 [a]	15.25 ± 0.42 [b]
24:1(n-9)	0.04 ± 0.02	0.11 ± 0.13	0.23 ± 0.37
16:0 DMA	0.02 ± 0.01	0.04 ± 0.05	0.12 ± 0.12
18:0 DMA	0.13 ± 0.14	0.06 ± 0.07	0.32 ± 0.18
18:1 DMA	0.52 ± 0.57	0.19 ± 0.38	0.38 ± 0.41

Values without a common superscript are significantly different from each other (ANOVA)

DMA: Dimethylacetal

Supplementary Materials, Table-S3

Pup retina Fatty Acid Composition (% of Total FA) Mean ± SD)

FA	Control Diet (n=6)	TAG-DHA diet (n=6)	LPC-DHA diet (n=6)
12:0	0.24 ± 0.18	0.02 ± 0.01	0.43 ± 0.45
14:0	0.43 ± 0.69	0.04 ± 0.07	0.23 ± 0.28
16:0	24.91 ± 1.98	23.03 ± 1.68	24.07 ± 2.07
16:1	1.16 ± 0.49	0.55 ± 0.29	0.91 ± 0.50
17:1	0.34 ± 0.35	0.06 ± 0.03	0.70 ± 0.41
18:0	21.30 ± 0.79	20.31 ± 1.35	19.73 ± 1.12
18:1 (n-9)	15.64 ± 4.97	20.07 ± 1.98	14.24 ± 4.12
18:1(n-7)	4.22 ± 0.60	4.52 ± 0.33	4.09 ± 0.61
18:2 (n-6)	1.83 ± 0.60	1.14 ± 0.11	2.16 ± 0.83
18:3 (n-6)	0.27 ± 0.29	0.04 ± 0.01	0.19 ± 0.13
18:3 (n-3)	0.32 ± 0.29	0.05 ± 0.05	0.21 ± 0.14
20:0	0.59 ± 0.15	0.49 ± 0.10	0.44 ± 0.07
20:1 (n-9)	1.84 ± 1.34	2.11 ± 1.08	1.35 ± 0.98
20:2 (n-6)	1.28 ± 1.08	1.00 ± 0.29	0.68 ± 0.48
20:3 (n-6)	0.57 ± 0.39	0.26 ± 0.19	0.61 ± 0.59
20:4 (n-6)	7.87 ± 0.92 [a]	9.54 ± 0.84 [b]	8.47 ± 0.98 [ab]
22:0	0.45 ± 0.44	0.09 ± 0.05	0.31 ± 0.24
20:5 (n-3)	0.70 ± 0.24	0.32 ± 0.16	0.52 ± 0.32
22:2	0.56 ± 0.43	0.23 ± 0.17	0.51 ± 0.36
22:3 (n-3)	1.80 ± 1.07	2.65 ± 0.24	1.24 ± 1.07
22:4 (n-6)	0.82 ± 0.68	0.06 ± 0.01	0.47 ± 0.30
22:5 (n-6)	0.77 ± 0.51	0.33 ± 0.15	0.47 ± 0.53
22:5 (n-3)	1.08 ± 0.86	0.19 ± 0.18	0.51 ± 0.55
22:6 (n-3)	11.11 ± 0.65 [a]	11.79 ± 0.19 [a]	17.30 ± 0.68 [b]
24:1	0.53 ± 0.86	0.06 ± 0.02	0.50 ± 0.40
16:0 DMA	0.16 ± 0.13	0.03 ± 0.02	0.12 ± 0.09
18:0 DMA	0.27 ± 0.16	3.09 ± 7.19	0.47 ± 0.32
18:1 DMA	0.76 ± 0.89	0.59 ± 0.61	0.32 ± 0.26

Values without a common superscript are significantly different from each other (ANOVA)

DMA: Dimethylacetal

Supplementary Materials, Table-S4

Pup liver Fatty Acid Composition (% of Total FA) Mean ± SD)

FA	Control Diet (n=6)	TAG-DHA diet (n=6)	LPC-DHA diet (n=6)
12:0	0.05 ± 0.03	0.08 ± 0.07	0.05 ± 0.03
14:0	1.47 ± 1.08	1.18 ± 0.33	1.46 ± 0.41
16:0	24.33 ± 3.04 [a]	27.31 ± 1.72 [a]	23.78 ± 1.66 [b]
16:1	0.98 ± 0.48	0.62 ± 0.32	0.86 ± 0.24
17:1	0.05 ± 0.03	0.07 ± 0.06	0.04 ± 0.03
18:0	12.34 ± 4.68	16.75 ± 3.00	12.24 ± 2.46
18:1 (n-9)	12.01 ± 3.51	9.16 ± 2.62	11.75 ± 2.68
18:1(n-7)	1.69 ± 0.34	1.66 ± 0.21	1.70 ± 0.17
18:2 (n-6)	19.71 ± 3.99	17.43 ± 2.38	20.06 ± 3.27
18:3 (n-6)	0.65 ± 0.29	0.53 ± 0.20	0.58 ± 0.11
18:3 (n-3)	0.46 ± 0.52	0.73 ± 0.65	0.32 ± 0.18
20:0	0.03 ± 0.01	0.08 ± 0.06	0.04 ± 0.05
20:1 (n-9)	0.23 ± 0.22	0.17 ± 0.18	0.15 ± 0.19
20:2 (n-6)	0.85 ± 0.75	1.13 ± 1.42	1.18 ± 1.26
20:3 (n-6)	1.06 ± 0.16	0.98 ± 0.15	1.00 ± 0.09
20:4 (n-6)	13.16 ± 0.83	14.79 ± 3.09	14.12 ± 1.26
22:0	0.05 ± 0.03	0.14 ± 0.11	0.07 ± 0.06
20:5 (n-3)	0.12 ± 0.07	0.11 ± 0.08	0.10 ± 0.14
22:2	0.04 ± 0.03	0.06 ± 0.09	0.04 ± 0.02
22:3 (n-3)	0.02 ± 0.01	0.05 ± 0.04	0.03 ± 0.03
22:4 (n-6)	0.03 ± 0.03	0.04 ± 0.03	0.03 ± 0.03
22:5 (n-6)	0.04 ± 0.03	0.08 ± 0.14	0.04 ± 0.06
22:5 (n-3)	0.02 ± 0.01	0.04 ± 0.04	0.04 ± 0.06
22:6 (n-3)	6.49 ± 0.18 [a]	6.61 ± 0.08 [a]	7.42 ± 0.36 [b]
24:1	0.04 ± 0.02	0.07 ± 0.05	0.05 ± 0.04
16:0 DMA	0.02 ± 0.02	0.07 ± 0.08	0.03 ± 0.02
18:0 DMA	4.01 ± 6.41	0.02 ± 0.03	0.02 ± 0.01
18:1 DMA	0.06 ± 0.03	0.07 ± 0.08	2.80 ± 6.60

Supplementary Materials, Table-S5

FA composition of female pups IWAT (% of Total FA) Mean ± SD

FA	Control Diet (n=7)	TAG-DHA diet (n=5)	LPC-DHA diet (n=5)
12:0	0.06 ± 0.03	0.05 ± 0.04	0.06 ± 0.04
14:0	9.69 ± 1.05	9.78 ± 1.31	10.41 ± 1.38
16:0	27.54 ± 0.94	28.01 ± 1.31	27.78 ± 1.65
16:1 (n-7)	4.16 ± 0.53	4.36 ± 0.36	4.48 ± 0.48
17:1(n-9)?	0.01 ± 0.01	0.01 ± 0.00	0.02 ± 0.02
18:0	2.01 ± 0.13	1.88 ± 0.19	1.84 ± 0.19
18:1 (n-9)	32.01 ± 2.27	29.98 ± 2.46	30.35 ± 3.10
18:1(n-7)	3.33 ± 0.39	3.11 ± 0.35	3.17 ± 0.39
18:2 (n-6)	17.86 ± 1.04	17.57 ± 0.44	17.54 ± 1.24
18:3 (n-6)	0.14 ± 0.09	0.23 ± 0.01	0.16 ± 0.09
18:3 (n-3)	0.15 ± 0.08	0.14 ± 0.10	0.14 ± 0.07
20:0	0.95 ± 0.04	0.79 ± 0.14	0.85 ± 0.14
20:1 (n-9)	0.02 ± 0.01	0.01 ± 0.00	0.01 ± 0.01
20:2 (n-6)	0.02 ± 0.03	0.02 ± 0.02	0.01 ± 0.01
20:3 (n-6)	0.29 ± 0.35	0.54 ± 0.32	0.41 ± 0.38
20:4 (n-6)	1.11 ± 0.48	1.16 ± 0.07	1.20 ± 0.11
22:0	0.02 ± 0.01	0.02 ± 0.01	0.02 ± 0.01
20:5 (n-3)	0.01 ± 0.02	0.02 ± 0.01	0.01 ± 0.01
22:2 (n-6)	0.03 ± 0.02	0.03 ± 0.03	0.03 ± 0.03
22:3 (n-3)	0.01 ± 0.00	0.02 ± 0.01	0.01 ± 0.01
22:4 (n-6)	0.08 ± 0.05	0.09 ± 0.04	0.08 ± 0.02
22:5 (n-6)	0.02 ± 0.01	0.02 ± 0.02	0.02 ± 0.01
22:5 (n-3)	0.02 ± 0.02	0.02 ± 0.01	0.02 ± 0.01
22:6 (n-3)	0.33 ± 0.26 [a]	2.00 ± 0.63 [b]	1.31 ± 0.56 [b]
24:1 (n-9)	0.04 ± 0.02	0.04 ± 0.03	0.03 ± 0.01
16:0 DMA	0.01 ± 0.01	0.01 ± 0.01	0.01 ± 0.01
18:0 DMA	0.02 ± 0.03	0.02 ± 0.02	0.00 ± 0.00
18:1 DMA	0.07 ± 0.07	0.07 ± 0.07	0.06 ± 0.04

Values without a common superscript are significantly different from each other (ANOVA)

DMA: Dimethylacetal

Supplementary Materials, Table-S6

FA composition of Male pups IWAT

FA	Control Diet (n=7)			TAG-DHA diet (n=5)			LPC-DHA diet (n=5)		
12:0	0.04	±	0.04	0.05	±	0.04	0.08	±	0.02
14:0	10.21	±	1.08	9.52	±	1.44	10.20	±	1.54
16:0	28.31	±	1.32	27.36	±	1.53	28.50	±	1.45
16:1	4.43	±	0.58	4.46	±	0.68	4.64	±	0.53
17:1	0.02	±	0.03	0.02	±	0.02	0.01	±	0.01
18:0	1.93	±	0.20	1.95	±	0.24	1.87	±	0.21
18:1 (n-9)	30.90	±	1.98	31.21	±	3.59	29.40	±	3.10
18:1(n-7)	3.13	±	0.26	3.29	±	0.53	3.05	±	0.43
18:2 (n-6)	18.20	±	0.77	17.12	±	1.05	17.67	±	0.39
18:3 (n-6)	0.15	±	0.10	0.22	±	0.02	0.24	±	0.01
18:3 (n-3)	0.13	±	0.02	0.18	±	0.08	0.16	±	0.08
20:0	0.87	±	0.13	0.87	±	0.15	0.79	±	0.13
20:1 (n-9)	0.02	±	0.01	0.01	±	0.00	0.01	±	0.01
20:2 (n-6)	0.01	±	0.01	0.02	±	0.03	0.02	±	0.03
20:3 (n-6)	0.29	±	0.35	0.52	±	0.32	0.60	±	0.38
20:4 (n-6)	0.71	±	0.64	1.18	±	0.13	1.14	±	0.07
22:0	0.02	±	0.01	0.02	±	0.00	0.04	±	0.02
20:5 (n-3)	0.02	±	0.01	0.02	±	0.01	0.01	±	0.01
22:2	0.04	±	0.03	0.02	±	0.01	0.04	±	0.02
22:3 (n-3)	0.02	±	0.01	0.01	±	0.01	0.01	±	0.01
22:4 (n-6)	0.06	±	0.04	0.08	±	0.04	0.09	±	0.04
22:5 (n-6)	0.03	±	0.02	0.03	±	0.01	0.02	±	0.03
22:5 (n-3)	0.02	±	0.01	0.02	±	0.01	0.03	±	0.02
22:6 (n-3)	0.33	±	0.15 [a]	1.72	±	0.49 [b]	1.26	±	0.46 [b]
24:1	0.03	±	0.01	0.03	±	0.02	0.03	±	0.02
16:0 DMA	0.01	±	0.01	0.01	±	0.01	0.01	±	0.00
18:0 DMA	0.03	±	0.05	0.01	±	0.01	0.01	±	0.01
18:1 DMA	0.05	±	0.04	0.06	±	0.04	0.07	±	0.05

Values without a common superscript are significantly different from each other (ANOVA)

DMA: Dimethylacetal
